# Transmission routes of African swine fever virus to domestic pigs: current knowledge and future research directions

**DOI:** 10.1136/vr.103593

**Published:** 2016-03-12

**Authors:** Claire Guinat, Andrey Gogin, Sandra Blome, Guenther Keil, Reiko Pollin, Dirk U. Pfeiffer, Linda Dixon

**Affiliations:** 1 Royal Veterinary College, Veterinary Epidemiology, Economics and Public Health Group, Hawkshead Lane, Hatfield, Hertfordshire AL9 7TA, UK; 2 The Pirbright Institute, Ash Road, Pirbright, Surrey GU24 0NF, UK; 3 European Food Safety Authority, Via Carlo Magno 1A, 43126 Parma, Italy; 4 Friedrich-Loeffler Institut, Sudufer 10, 17493 Greifswald-Insel Riems, Germany

## Abstract

African swine fever (ASF) is a major threat to the pig industry in Europe. Since 2007, ASF outbreaks have been ongoing in the Caucasus, Eastern Europe and the Baltic countries, causing severe economic losses for many pig farmers and pork producers. In addition, the number of ASF cases in wild boar populations has dramatically increased over the past few years. Evidence supports direct contact with infectious domestic pigs and wild boars, and consumption of contaminated feed, as the main transmission routes of ASF virus (ASFV) to domestic pigs. However, significant knowledge gaps highlight the urgent need for research to investigate the dynamics of indirect transmission via the environment, the minimal infective doses for contaminated feed ingestion, the probability of effective contacts between infectious wild boars and domestic pigs, the potential for recovered animals to become carriers and a reservoir for transmission, the potential virus persistence within wild boar populations and the influence of human behaviour for the spread of ASFV. This will provide an improved scientific basis to optimise current interventions and develop new tools and strategies to reduce the risk of ASFV transmission to domestic pigs.

AFRICAN swine fever (ASF) is one of the pig diseases with the highest mortality. Many ASF virus (ASFV) strains result in the death of almost 100 per cent of infected pigs. In addition to the impact on animal health and people's livelihoods, the disease can have a major impact on global trade in pigs and pork products and is a threat to global food security (EFSA 2014). ASFV is endemic in most of sub-Saharan Africa and Sardinia and, since the first case in Georgia in 2007, has been spreading through the Caucasus, Eastern Europe and the Baltic countries (see geographical regions determined according to the United Nations Statistics Division: http://unstats.un.org/unsd/methods/m49/m49regin.htm. (Caucasus includes Georgia, Armenia and Azerbaijan; Eastern Europe includes Belarus, Bulgaria, Czech Republic, Hungary, Moldova, Poland, Romania, Russia, Slovakia and Ukraine; and the Baltic states includes Estonia, Latvia and Lithuania.) ASFV is generally spread by contact with infectious animals and fomites, ingestion of contaminated pig products and tick bites. However, ASFV transmission and maintenance varies substantially between countries. In sub-Saharan Africa, the disease is endemic and circulates through a cycle of infection involving domestic pigs, bushpigs (*Potamochoerus larvatus*), warthogs (*Phacochoerus aethiopicus*) and soft ticks of the *Ornithodoros* species (Plowright and others 1994). In areas of the Caucasus, Eastern Europe and the Baltic countries, the disease circulates among domestic pigs (*Sus scrofa domesticus*) and European wild boars (*Sus scrofa*), causing similar clinical signs and mortality in both populations (Gogin and others 2013).

ASFV is likely to have been introduced into Georgia via imports of contaminated pig products from Eastern Africa or Madagascar (Rowlands and others 2008). Since then, the disease has subsequently spread to Eastern Europe and the Baltic countries, most likely through movements of infected wild boars and domestic pigs, and contaminated pig products. As of October 2015, more than 750,000 dead or culled domestic pigs and 1300 infected European wild boars due to ASF have been reported to the World Organisation for Animal Health (OIE) in these countries (OIE 2015). Over the past few months, increased numbers of outbreaks have been reported in wild boar populations in the Baltic countries, in total 878, compared to 39 in domestic pig populations (OIE 2015). ASFV transmission to domestic pigs is likely to be influenced by social attitudes and economic considerations. A recent survey showed that many farmers from Russia, Bulgaria and Germany believe that reporting ASF would adversely affect their reputation and they therefore prefer to control the outbreak themselves, without the involvement of veterinary services (Vergne and others 2014). This would explain why remains of infected pigs were discovered in Russia, probably hidden by pig owners (Gogin and others 2013, Oganesyan and others 2013). Accordingly, when financial compensation is lacking, farmers are suspected to sell animals or their products to reduce economic losses before disease confirmation (FAO 2013).

It is important that ASF-free areas are protected against the introduction of the disease particularly since no vaccines or treatments are available to aid control. Control and prevention programmes have been developed for ASF (EC 2002, 2003, 2013, EU 2014), providing recommendations at many levels, from pig holdings to government bodies. However, ASF continues to spread in several parts of the Caucasus, Eastern Europe and the Baltic countries. Containing the ongoing epidemic remains a challenge in these countries due to the high stability of the virus in meat products and the environment, the potential legal and illegal movements of pigs and their products, the number of low biosecurity farms, the lack of disease awareness, the similarity of clinical signs to other pig diseases, the practice of swill feeding, and the increased numbers of infected wild boar and their interactions with susceptible domestic pigs (EFSA 2014). Thus, the Food and Agriculture Organization of the United Nations (FAO) has stressed the importance of continuing international efforts to improve the understanding of ASFV transmission and prevent further global spread (FAO 2011).

This review summarises current knowledge on the transmission routes of ASFV to domestic pigs, with a focus on the current situation in the Caucasus, Eastern Europe and the Baltic countries. We highlight the most significant knowledge gaps, where data are lacking, with the view to identify future research priorities. Outcomes will be used to develop and optimise control policies to prevent and control further spread of ASF.

## Pig-to-pig transmission

Recent experimental studies have provided the range of infectious blood, excretions and secretions from pigs. Up to 10^9^ 50 per cent haemadsorbing doses per ml (HAD_50_/ml) could be detected in blood and up to 10^5^ HAD_50_/ml in saliva, urine or faeces (Table 1), following infection of pigs with highly virulent ASFV strains currently circulating in Lithuania (2014), Georgia (2007) and Russia (2013) (Guinat and others 2014, Gallardo and others 2015a, Vlasova and others 2015). Similar values were observed following infection of pigs with moderately virulent ASFV strains that were circulating during past ASF outbreaks in the Netherlands (1986), Portugal (1968) and Malta (1978) (Wilkinson and others 1981, de Carvalho Ferreira and others 2012, Gallardo and others 2015b). Some pigs (30 to 50 per cent) recover from infection with these moderately virulent isolates and viral DNA could also be detected intermittently in air samples from the acute phase and during a longer persistence phase from four to 70 days post-infection. Detection in air samples was significantly associated with the intermittent detection of virus DNA in faeces (de Carvalho Ferreira and others 2013b).

**TABLE 1:** Quantification of African swine fever virus (ASFV) in blood, secretions and excretions of infected domestic pigs with currently circulating strains in Caucasus, Eastern Europe and the Baltic countries

Sample type	ASFV strain	Inoculation	Maximum of virus titres detected	References
Blood	Lithuania LT14/1490 isolated from wild boar	Intramuscular 10 HAD_50_/ml	10^6.4^ to 10^8.7^ HAD_50_/ml at 6 dpi	Gallardo and others 2015a
		Contact	10^6.4^ to 10^8.7^7 HAD_50_/ml at 14 dpi	
	Georgia 2007/1 isolated from domestic pig	Intramuscular 10^2^ HAD_50_/ml	10^6^ to 10^8^ HAD_50_/ml from 5 dpi	Guinat and others 2014
		Contact	10^6^ to 10^8^ HAD_50_/ml from 10 dpi	
	Russia Kashino 04/13 isolated from wild boar	Intranasal 5×10^3^ HAD_50_/ml	10^7.5^ HAD_50_/ml at 7 dpi	Vlasova and others 2015
		Intranasal 50 HAD_50_/ml	10^6.5^ to 10^7.5^ HAD_50_/ml from 7 dpi	
		Contact	10^6.5^ to 10^7^ HAD_50_/ml from 15 dpi	
	Russia Boguchary 06/13 isolated from domestic pig	Intranasal 5×10^3^ HAD_50_/ml	10^6.5^ to 10^7.5^ HAD_50_/ml from 9 dpi	Vlasova and others 2015
		Intranasal 50 HAD_50_/ml	10^6.5^ to 10^7^ HAD_50_/ml from 5 dpi	
		Contact	10^7^ HAD_50_/ml at 13 dpi	
	Russia K 08/13 isolated from wild boar	Intramuscular 5×10^3^ HAD_50_/ml	10^6.5^ to 10^7^ HAD_50_/ml from 7 dpi	Vlasova and others 2015
		Intramuscular 50 HAD_50_/ml	10^6.5^ to 10^7^ HAD_50_/ml from 9 dpi	
Nasal fluid	Georgia 2007/1 isolated from domestic pig	Intramuscular 10^2^ HAD_50_/ml	Intermittent detection, 10^2^ to 10^4^ HAD_50_/ml from 6 dpi	Guinat and others 2014
		Contact	Intermittent detection, 10 to 10^2^ HAD_50_/ml from 7 dpi	
Rectal fluid	Georgia 2007/1 isolated from domestic pig	Intramuscular 10^2^ HAD_50_/ml	Intermittent detection, 10 to 10^2^ HAD_50_/ml from 5 dpi	Guinat and others 2014
		Contact	Intermittent detection, 10 to 10^2^ HAD_50_/ml from 12 dpi	

dpi Day post-infection, HAD_50_/ml 50 per cent haemadsorbing doses per ml

Several experimental studies demonstrated that direct contact with infectious domestic pigs is an effective mechanism of ASFV transmission. Susceptible pigs housed together with pigs infected with the ASFV strains from Lithuania and Georgia became infected by direct contact after one to nine days post-exposure (dpe) (Gallardo and others 2015a, Guinat and others 2016). When contact pigs were separated from the infectious pigs by solid partitions to prevent direct pig contact between pens, the transmission occurred after six to 15 dpe (Guinat and others 2014). Similar effective transmission contacts were obtained using highly virulent ASFV strains from Malawi (1962) and Tanzania (1970) (Greig and Plowright 1970, Howey and others 2013). Transmission using a low virulence ASFV strain from Portugal (1988) was demonstrated between domestic pigs but was less efficient (from 42 to 50 per cent of the contact pigs became infected) than using a highly virulent ASFV strain (100 per cent), probably due to the fact that low and sporadic viremia was only developed by two (out of 11) infected donor pigs (Boinas and others 2004).

Authors have also quantified the transmission dynamics for different ASFV strains under field and experimental conditions. The basic reproduction number (R_0_, eg, the average number of newly infected animals caused by one infectious animal) was estimated for the Malta ASFV strain at 18.0 (95 per cent confidence interval [CI]: 6.9 to 46.9) (de Carvalho Ferreira and others 2013a) and for the Georgia and Russia ASFV strains at 1.4 (95 per cent CI 0.6 to 2.4), 2.8 (95 per cent CI 1.3 to 4.8) (Guinat and others 2016) and 9.8 (95 per cent CI 3.9 to 15.6) (Gulenkin and others 2011), depending on the transmission scenarios (Table 2). Comparison of these estimates remains difficult due to differences in estimation methods, such as assumptions in relation to infection and infectiousness markers and diagnostic tools.

**TABLE 2:** Quantification of African swine fever virus (ASFV) transmission among domestic pigs and wild boar under experimental and field conditions

Transmission scenario		ASFV strain	Latent period duration (days)	Infectious period duration (days)	Basic reproduction number (95 per cent confidence interval [CI])	References
*Experimental studies*						
Pig-to-pig	Direct	Georgia 2007	4	3 to 6	2.8 (1.3 to 4.8)	Guinat and others 2016
				3 to 14	5.3 (1.7 to 10.3)	
	Indirect			3 to 6	1.4 (0.6 to 2.4)	
				3 to 14	2.5 (0.8 to 5.2)	
Wild boar-to-wild boar	Direct	Armenia 2008	4	2 to 9	6.1 (0.6 to 14.5)	Pietschmann and others 2015
Wild boar-to-pig	Direct				5.0 (1.4 to 10.7)	
	Indirect				0.5 (0.1 to 1.3)	
Pig-to-pig	Direct	Malta 1978	3 to 6	4 to 10	18.0 (6.9 to 46.9)	de Carvalho Ferreira and others 2013a
*Field studies*						
Wild boar-to-wild boar	Between-group	Russia	-	-	1.58 (1.1 to 3.8)	Iglesias and others 2015
Pig-to-pig	Within-farm	Russia	15	5	9.8 (3.9 to 15.6)	Gulenkin and others 2011

Knowledge is lacking concerning the possible existence of a pig-carrier state, that is, a pig shedding ASFV without showing any clinical signs and being a potential source of ASFV infection, after infection with the strains circulating in the Caucasus, Eastern Europe and the Baltic countries. After infection with the ASFV strains from Lithuania, Georgia and Russia, pigs mainly developed the acute form of the disease (Guinat and others 2014, Gallardo and others 2015a, Vlasova and others 2015). They became generally infectious three to five days post-inoculation (dpin) and did not survive for more than seven to 13 dpin. Chronic forms of the disease have been only observed in pigs experimentally infected with reduced virulence ASFV strains from past ASF outbreaks in Europe (Wilkinson and others 1981, de Carvalho Ferreira and others 2012, Gallardo and others 2015b). However, two recent studies suggested that some pigs infected with the ASFV isolated from wild boars in Russia and Lithuania could develop longer courses of infection (up to 21 dpin) (Vlasova and others 2015) or remained asymptomatic (Gallardo and others 2015a). Thus, this may indicate the development of a carrier state in domestic pigs, although the amount of time for ASFV to evolve towards lower virulence in the Caucasus, Eastern Europe and the Baltic countries has been relatively short, compared to ASFV strains that have been circulating for decades during past ASF outbreaks in Europe and Africa.

## Feed-to-pig transmission

Studies have provided the range of possible contaminated pig products that could be consumed by susceptible domestic pigs. ASFV can persist for months in pork meat, fat and skin and in different types of pork products, such as sausages and salami stored under experimental conditions at negative and room temperature (McKercher and others 1978, Mebus and others 1993, 1997). In the field, ASFV has been detected in meat products in Russia (Gogin and others 2013) and in Latvia (six out of 42 samples of meat products were positive for ASFV genome) (EC 2014a) close to the border with Belarus. This emphasises the potential relevance for this route of transmission. Therefore, swill feeding, a common practice in the traditional pig production systems with free-ranging and backyard pigs globally (Costard and others 2009, Kagira and others 2010, Phengsavanh and others 2010) could play an important role in the ASFV transmission to domestic pigs. This may explain why most of the ASF outbreaks in Russia have been described in free-ranging and backyard farms before occurring in large commercial farms (Gogin and others 2013). Recent epidemiological investigations in Latvia and Lithuania have also suggested that fresh grass and seeds potentially contaminated by secretions from infectious wild boars (EC 2014a) are possible sources of infection for backyard farms.

However, we still know relatively little about factors that are important for ASFV transmission through contaminated feed. Transmission has been experimentally demonstrated with contaminated milk (Greig 1972). In this study, the oral median infectious dose (ID_50_) of a highly virulent Tanzania ASFV strain was determined at 10^5.4^ HAD_50_/ml. One study also showed that domestic pigs were infected when consuming faeces and urine contaminated with a virulent Kenya ASFV strain, although this failed when consuming contaminated sweet potatoes or bananas (Montgomery 1921). It has been reported that infection by ingestion of pig tissues contaminated with this strain required a high dose of virus (at least 10^5^ HAD_50_/ml) to effectively infect pigs (Heuschele 1967). Other authors determined the intranasal median ID_50_ of a highly virulent East Africa ASFV strain as 10^2.9^ HAD_50_/ml, although domestic pigs were not infected by consuming food contaminated with a higher dose of the same virus (Parker and others 1969), suggesting there might be different infection doses depending on whether ASFV is contained in food or directly orally inoculated. Several studies have investigated the relationship between route of inoculation, infectious dose and virulence level. Authors reported that the nasal route resulted in higher ASF incidence than the oral route when using a lower infectious dose, suggesting a more permissive infection route by inhalation than by ingestion (Howey and others 2013). The nasal/oral ID_50_ was found to be higher (by approximately 10 times) using an ASFV strain of high virulence than strains of reduced virulence (McVicar 1984). This was not confirmed in a recent study in which a very low dose exposure (by inhalation of 3 HAD_50_/ml) of a highly virulent ASFV strain from Armenia (2008) resulted in the same clinical course as high dose direct contact infection (Pietschmann and others 2015). Lower nasal ID_50_ were also suggested to be related to isolates with higher virulence (de Carvalho Ferreira and others 2012).

## Wild boar-to-pig transmission

Experimental studies demonstrated that wild boars were as susceptible as domestic pigs to ASFV infection using highly virulent ASFV strains from Armenia (2008) and Chechnya (2009) (Gabriel and others 2011, Blome and others 2012, Pietschmann and others 2015). Oral (dose of 10^6^ TCID_50_), nasal (dose of 3 to 25 HAD_50_/ml) and intramuscular (dose of 10^3^ HAD_50_/ml) infections resulted in 100 per cent mortality. Wild boars developed non-specific clinical signs, similar to those observed in domestic pigs, including fever, loss of appetite, diarrhoea and lethargy and died within seven to nine days, regardless of age or sex.

Two recent experimental studies indicated that direct contact with infectious wild boars is an effective ASFV transmission route to domestic pigs. Susceptible pigs housed in direct contact with infected wild boars with the ASFV strains from Armenia or Chechnya became infectious after six to 12 dpe (Gabriel and others 2011, Pietschmann and others 2015). When susceptible pigs were separated from the infectious wild boars in an adjacent pen to prevent direct contact, the transmission occurred after 21 dpe (Pietschmann and others 2015). The author estimated the R_0_ between groups of wild boars and domestic pigs using the Armenia ASFV strain (Pietschmann and others 2015) at 5.0 (95 per cent CI 1.4 to 10.7) and 0.5 (95 per cent CI 0.1 to 1.3) in direct and indirect contact scenarios, respectively (Table 2). During ASF outbreaks in Russia, the dynamics of ASFV transmission between groups of wild boars was recently quantified by the R_0_ at 1.58 (95 per cent CI 1.1 to 3.8) (Table 2) (Iglesias and others 2015).

There are several field observations about the possible contribution of infected wild boars to the spread of ASFV to domestic pigs. In Russia, some ASF cases were primarily detected in wild boars before being observed in domestic pigs, and the death of wild boars caused by ASF was observed in the vicinity of ASF-affected farms (Gogin and others 2013). A recent study has demonstrated that ASF cases in domestic pigs and wild boars were spatially correlated in the north west areas of Russia (Vergne and others 2015). High numbers of infected wild boar carcases were found close to national borders, for example, in Russia close to Georgia, in Poland and Lithuania close to Belarus and in Ukraine close to Russia (Gallardo and others 2014). One explanation proffered for this is that the recent attempts to reduce the number of wild boars in the region using intensive hunting practices have induced significant changes in the daily scavenging distance around the home of wild boar populations as they are trying to escape (Sodeikat and Pohlmeyer 2007, Thurfjell and others 2013), and these potentially facilitated ASFV spread over longer distances.

ASFV is therefore likely to transmit between wild boars by contact with infectious wild boars, infectious free-ranging pigs or carcases of infected pigs or wild boars improperly disposed of by farmers or hunters. However, it remains unclear whether ASFV can be sustained in these wild boar populations. For example, in contrast to results from north-west Russia, recent analyses showed that there was no space-time interactions among ASF cases in wild boars in south-west areas of Russia, suggesting the limited persistence of ASFV in wild boar populations (Lange and others 2014).

## Fomites-to-pig transmission

Studies have provided the range of possible environmental sources for ASFV transmission to domestic pigs. ASFV can persist for weeks in blood, faeces and urine excreted in the environment by infected pigs (Montgomery 1921, Plowright and Parker 1967, Haas and others 1995, Turner and Williams 1999). Periods of ASFV survival were estimated in faeces and urine contaminated with the highly virulent Georgia strain, as up to eight and 15 days at 4°C, respectively and five days at 21°C (Davies and others 2015).

However, infection of domestic pigs by contact with contaminated fomites has never been clearly demonstrated. A number of ASF outbreaks that occurred in large commercial farms in Russia and Lithuania have been explained by potential gaps in terms of compliance with the biosecurity rules, such as improper disinfection of clothing and boots, or contaminated food brought onto the premises (Gogin and others 2013, Oganesyan and others 2013, EC 2014b). Farmers that are hunting might also increase the risk of ASFV introduction into pig farms, particularly through the handling of potentially infected wild boar carcases.

## Tick-to-pig transmission

Soft ticks of the genus *Ornithodoros* have been identified as competent vectors of ASFV to domestic pigs (Sanchez Botija 1962), although involvement in the Caucasus, Eastern Europe and the Baltic countries is unlikely. In Eastern and Southern Africa, ASFV is maintained in a transmission cycle occurring between warthogs (*Phacochoerus africanus*) and *Ornithodoros moubata* complex ticks that live in their burrows (Plowright and others 1969, Thomson 1985).

To date, the presence of *Ornithodoros erraticus* complex ticks have been historically reported in the Caucasus countries and in Russia (Manzano-Román and others 2012) but their role in transmission of ASFV has not been defined. One experimental study has, however, indicated that ASFV Georgia strain was able to replicate in *O erraticus* ticks that are commonly found in Southern Europe and remain for at least 12 weeks (Diaz and others 2012). However, ASFV was not able to replicate in hard ticks (*Ixodes ricinus* and *Dermacentor reticulatus*), that are also commonly found in Europe, suggesting a limited vector competence for this tick family (de Carvalho Ferreira and others 2014).

*Stomoxys* flies have been shown to be experimentally competent for mechanically transmitting ASFV to domestic pigs for a limited time (Mellor and others 1987, Baldacchino and others 2013). However, flies collected on ASF-affected farms in Lithuania tested negative for ASFV (EC 2014b). ASFV has also been detected in *Haematopinus suis*, swine lice prevalent in temperate regions, collected from experimentally infected domestic pigs (Sanchez Botija and Badiola 1966).

Blood samples from other live animals, such as rodents and birds, have been collected from ASF-affected farms in Lithuania (EC 2014b) and Russia (EFSA 2014) but tested negative for ASFV.

## Research priorities

Important progress has been made over the past few years regarding the understanding of the important sources for ASFV transmission in the Caucasus, Eastern Europe and the Baltic countries. The research priorities are summarised in Table 3. Transmission of ASFV to domestic pigs has been mainly demonstrated by the contact of infected pigs via infectious body fluids as well as by aerosol over short distances between pens. Current knowledge also shows that the transmission of ASFV is possible by the ingestion of contaminated feed. However, more research is needed to clarify which are the minimum infection doses for domestic pigs when consuming feed containing unprocessed infected pig tissues. Moreover, this review highlighted that under experimental conditions, ASFV infection and transmission from wild boars to domestic pigs may be very similar to what has been observed for the pig-to-pig transmission scenarios. In the field, the duration and extent of exposure will have a major role in determining transmission from wild boars to domestic pigs. The key may lie in characterising the interfaces between wild boars and domestic pigs and determining the probability of effective contact between the two populations. Doubts still exist with respect to potential reduction in the virulence of ASFV strains circulating in the Caucasus, Eastern Europe and the Baltic countries and the possibility of domestic pigs or wild boars developing chronic infections, recovering and becoming carriers. This identifies a need for further research on the evolution, molecular epidemiology, pathology and immunology of ASFV infections. Knowledge concerning the role of fomites (surfaces of vehicles, equipment and animal worker clothing) in ASFV transmission is also lacking and further investigation is needed. Finally, social research studies should be developed to further understand the drivers for eradicating the disease and complying with biosecurity regulations, as this is crucial for effective control policies.

**TABLE 3:** Research priorities to improve African swine fever control in Caucasus, Eastern Europe and the Baltic countries

Type of studies	Research priorities
Laboratory-based studies	Further develop animal infection trials to investigate the effects of different strains, doses and routes of exposure, including by ingestion of contaminated feed or infected ticks
	Develop better diagnostic tests for environmental samples, including bedding and air
	Evaluate the potential of disease virulence evolution
Disease modeling studies	Develop transmission models for simulating disease spread within and between farms and assess the cost-benefit of alternative mitigation strategies (such as use of risk-based surveillance, different radii and duration for the surveillance zones, etc)
	Model the disease transmission using mortality data and clinical signs collected in different infected farm settings
	Develop transmission models for simulating disease spread between domestic pigs and wild boars and between wild boars populations
Field studies	Develop new approaches to better understand the potential contacts between domestic pigs and wild boar populations
	Develop improved methods to collect field data on wild boar population dynamics, movement patterns and disease prevalence
	Develop more sensitive methods to use for sampling of environmental materials (such as equipment, clothing, vehicles, etc)
	Integrate animal data (such as mortality, time period of clinical signs, etc) and human data (such as movements of animal workers and trucks) to explore other potential transmission pathways
	Further conduct field observations to assess vector distribution and competence
Social studies	Conduct in depth studies of human behaviour patterns to evaluate pig farm practices, awareness of disease epidemiology and obstacles to disease suspicion and reporting
